# Clinical Experience with the Single Dose of the CM Potency

**Published:** 1894-12

**Authors:** Erastus E. Case

**Affiliations:** Hartford, Conn.


### CLINICAL EXPERIENCE WITH THE SINGLE DOSE OF THE CM POTENCY.

Erastus E. Case, M. D., Hartford, Conn.

### I. Æthusa-cynapium.

October 2d, 1894.—A dark-haired girl eleven months of age. The mother of the child was at the end of the second week with typhoid fever when she was delivered, and died a few days later under allopathic treatment. The child has always been puny and suffered from indigestion, also been dosed by drugs. In August she nearly died from cholera infantum; was probably kept alive by the use of artificial digestive powders, but has not improved.

Stools frequent, fluid, green, containing curds, and with much colic, pain preceding and during the evacuations.

Anorexia; sometimes gagging and vomiting of a greenish fluid when the bottle is offered her.

Sleepless, or sleeps in short naps, with restlessness or grating of the teeth.

Her diet of cow's milk (diluted) was not changed, but the peptogenic powders were forbidden. One powder Æthusa-cyn.$^{cm}$ (F.).

October 4th.—The number of stools per day has decreased from twelve or more to four or five. The curds are less in number and size. The color is now usually yellow; consistency unchanged. The odor, which was slight, is now unbearably offensive. No medicine.

October 7th.—Now there are not more than two or three stools daily, yellow, with normal consistency, rarely with any curds.

The improvement continued.

### II. Daphne-indica.

August 8th, 1894.—An obese traveling salesman (weight 250 pounds), of forty-three years.

Swaying sensation in the head.

Sensation as if the head was separated from the body (constant).

Sensation as if the vertex was screwed up in a vise.

Often, while driving a horse, the arms feel as if separate from the body, yet they are fully under control of the will. One powder, Daphne-ind.$^{cm}$ (F.).

October 10th.—He has experienced complete relief from the symptoms until within a few days. One powder, Daphne-ind.$^{cm}$ (F.).

November 5th.—None of the troubles since last prescription.

### III. Belladonna.

August 7th, 1894.—A black-haired man, sixty-eight years old, retired seaman, subject to a chronic cough. While straining at stool, an hour ago, he was taken with such severe pain in the region of the appendix vermiformis that he had to be carried bodily to the bed. A few weeks ago a nephew was attacked in nearly the same way, dosed with Morphine by our local appendivermiformophobist, and cut open within twenty-four hours. He escaped with his life, but minus $300 of his hard-earned cash. He commands: "Give me a hypodermic injection." I reply: "I never use them," and ask for symptoms. He refuses to tell any, declaring that I must give something at once to either kill or relieve him. "Sir, I did not come here to kill you or to stupefy you, but to cure you, which I will do if you will tell me your symptoms."

Severe, cutting, lancinating pains extending from the ileocœcal region outward toward the hip-joint.

So tender to touch that he cannot endure the weight of hot compresses.

Some relief from heat.

Slight swelling of the part affected.

His face is already pinched and haggard from the intensity of the pain. One powder Belladonna$^{cm}$ (F.).

He was asleep within fifteen minutes, and awoke in two hours free from pain and soreness. On August 10th there was a recurrence of the pain, but less severe. It was again relieved by a dose of Belladonna$^{cm}$. Since then he has been free from the trouble.

### IV. Sabina.

August 5th, 1894.—A brown-haired woman, aged thirty-four years, German, mother of four children. Profuse menstruation began yesterday. Two hours ago paroxysmal pain came on, extending from the pubic bone to the sacrum. It is so agonizing as to extort groans and tears from a woman who has been in the habit of bearing pain with fortitude. One powder Sabina$^{cm}$ (F.).

A pain soon followed the administration of the remedy, the most severe of all, the patient affirmed, and that was the last one. The flow, which had been like a hemorrhage up to that time, bright and fluid, became like a normal catamenial discharge.

### V. Benzoic-acid.

April 29th, 1894.—A woman of seventy-one years, dark-haired, thin, nervous temperament. Left-sided hemiplegia came on six weeks ago, from which she is recovering, having now some use of the hand and able to sit up. She has been troubled by urinary strangury for some months, and since this illness it has caused her and the attendants much annoyance.

Urine scanty, very offensive; no relief from the urging even after it is voided. Several remedies have been given without effect. One powder Benzoic-acid$^{cm}$ (F.).

This gave her permanent relief so long as she was a resident of Hartford (four months).

### VI. Graphites.

October 4th, 1894.—A black-haired woman, forty-four years old. On September 20th a crack came in the left corner of the mouth. Moisture from it formed a yellowish crust. Sores now cover the chin and are scattered over the whole face. A blister first appears, with heat and itching. This breaks and a crust follows. There is much itching, and if the crusts are removed a sticky moisture exudes, which soon renews the yellow covering.

Menses irregular, usually late and scanty.

Leucorrhœa profuse, milky white, causing itching.

Always awakes lying upon the back, with pain in the sacrum. It hurts her there to turn over. Relieved after arising.

Throbbing pain over the eyes, with soreness of the ocular muscles and dark circles under the eyes; worse from over-exertion or excitement, while lying down, usually before or after the catamenia; better in cold, open air, from hot applications.

Feet cold and dry. One powder Graphites$^{cm}$ (F.).

October 11th.—The crusts came off on the 8th, leaving a dry surface on their site. Some thin scales are now on the chin only. Since the prescription she has had a severe catarrhal cold, the explanation of which need not be given to a genuine homœopathist.

October 18th.—Face entirely well; health greatly improved.

### VII. Bufo-cinereus.

August 6th, 1894.—A girl of ten years, brown-haired, nervous temperament. She had scarlatina a year ago, and has not been strong since.

Epistaxis nearly every day for some weeks, sometimes several attacks daily, usually with a flushed face, often with heat and pain in the forehead, which are relieved by the nose-bleed.

Easy perspiration, which is apt to be offensive, especially upon the feet. One powder Bufo-cinereus$^{cm}$ (F.).

Cured at once, and her health improved wonderfully.

### VIII. Cannabis-indica.

December 25th, 1893.—A man, seventy-two years old, tall, stooping; retired civil engineer. Frequent micturition. During urination, pain in the glans penis, also a thrill, which goes all over him, even to the hands.

Sensation as if a ball were in the anus when sitting. One powder Cannabis-indica$^{cm}$ (F.). Immediate relief followed.

### IX. Lachesis.

September 24th, 1894.—A black-haired machinist, æt. forty-nine years. Eleven weeks ago he accidentally bruised the inside of the left lower leg. He has three foul ulcers, where the skin was scraped off; the largest is one inch in diameter, the others about half that size. He has been under the care of a "modern homœopathist."

The skin around the ulcers has an unhealthy, bluish color.

Stinging pains in the ulcers when standing.

Fifteen years ago he had for months a sore on the top of his left foot, which was called erysipelas.

When eleven years of age he was hit by a stone in the back of the head. Inflammation (called erysipelas) of the scalp and neck, with delirium, followed for three months.

He is subject to facial erysipelas, left-sided, bluish-colored, and blistered. One powder Lachesis$^{cm}$ (F.).

October 15th.—The largest ulcer has decreased in size nearly one-half, the others are entirely healed. The color of the surrounding skin is now quite natural.

October 30th.—The limb is now sound.

### X. Bryonia-alba.

August 19th, 1894.—A dark-haired young man of eighteen years, clerk in fruit store.

Diarrhœa since the 15th; stools brown, fluid, with burning and some tenesmus; frequent by night and day.

Chilly yesterday and fever since then. Temperature 104°.

Tongue white; thirst for much cold water.

Severe pain in the occiput and back, relieved by lying on the back.

Vertigo and increased pain in the head from an erect position.

Soreness about the ileo-cœcal valve; also a gurgling of gas is perceptible to the touch when the hand is pressed upon that region.

Dreams and talks about his business during sleep.

Some delirium when awaking from sleep. One powder Bryonia$^{cm}$ (F.).

August 26th.—The patient has shown a steady improvement through the first week of the fever. The stools became less frequent and pasty. Sleep became more quiet and restful, with less delirium. The temperature decreased from day to day until it reached 101.4° yesterday morning. Perspiration first appeared on the 23d. The lenticular maculæ, characteristic of typhoid fever, are now appearing upon the abdomen. Temperature this morning, the beginning of the second week, 100.2°.

September 1st.—Improvement has been steady through the second week. The temperature diminished until it reached 98.5° yesterday morning without an evening rise.

No complications or increase in severity of the symptoms in the course of the disease called for another dose of the same or any other remedy. He sat up on September 2d, and began light work on the 10th of the month.

